# Association of Mild Anemia with Cognitive, Functional, Mood and Quality of Life Outcomes in the Elderly: The “Health and Anemia” Study

**DOI:** 10.1371/journal.pone.0001920

**Published:** 2008-04-02

**Authors:** Ugo Lucca, Mauro Tettamanti, Paola Mosconi, Giovanni Apolone, Francesca Gandini, Alessandro Nobili, Maria Vittoria Tallone, Paolo Detoma, Adriano Giacomin, Mario Clerico, Patrizia Tempia, Adriano Guala, Gilberto Fasolo, Emma Riva

**Affiliations:** 1 Laboratory of Geriatric Neuropsychiatry, Istituto di Ricerche Farmacologiche “Mario Negri”, Milano, Italy; 2 Laboratory for Medical Research & Consumer Involvement, Istituto di Ricerche Farmacologiche “Mario Negri”, Milano, Italy; 3 Laboratory of Translational and Outcome Research in Oncology, Istituto di Ricerche Farmacologiche “Mario Negri”, Milano, Italy; 4 Laboratory of Analysis, Ospedale degli Infermi, Biella, Italy; 5 County Cancer Registry, Local Health Authority, ASL12, Biella, Italy; 6 Department of Oncology, Ospedale degli Infermi, Biella, Italy; 7 Department of Medicine & Geriatrics, Ospedale degli Infermi, Biella, Italy; 8 Community Medicine, Local Health Authority, ASL12, Biella, Italy; James Cook University, Australia

## Abstract

**Background:**

In the elderly persons, hemoglobin concentrations slightly below the lower limit of normal are common, but scant evidence is available on their relationship with significant health indicators. The objective of the present study was to cross-sectionally investigate the association of mild grade anemia with cognitive, functional, mood, and quality of life (QoL) variables in community-dwelling elderly persons.

**Methods:**

Among the 4,068 eligible individuals aged 65–84 years, all persons with mild anemia (n = 170) and a randomly selected sample of non-anemic controls (n = 547) were included in the study. Anemia was defined according to World Health Organization (WHO) criteria and mild grade anemia was defined as a hemoglobin concentration between 10.0 and 11.9 g/dL in women and between 10.0 and 12.9 g/dL in men. Cognition and functional status were assessed using measures of selective attention, episodic memory, cognitive flexibility and instrumental and basic activities of daily living. Mood and QoL were evaluated by means of the Geriatric Depression Scale-10, the Short-Form health survey (SF-12), and the Functional Assessment of Cancer Therapy-Anemia.

**Results:**

In univariate analyses, mild anemic elderly persons had significantly worse results on almost all cognitive, functional, mood, and QoL measures. In multivariable logistic regressions, after adjustment for a large number of demographic and clinical confounders, mild anemia remained significantly associated with measures of selective attention and disease-specific QoL (all fully adjusted *p*<.046). When the lower limit of normal hemoglobin concentration according to WHO criteria was raised to define anemia (+0.2 g/dL), differences between mild anemic and non anemic elderly persons tended to increase on almost every variable.

**Conclusions:**

Cross-sectionally, mild grade anemia was independently associated with worse selective attention performance and disease-specific QoL ratings.

## Introduction

Mean blood concentrations of hemoglobin progressively decline with aging [1]. In the elderly persons, hemoglobin concentrations slightly below the lower limit of normal are common and are usually viewed by the physician as having no clinical significance or as a chronic disease marker with no independent effect on health. In recent years however, anemia has been increasingly shown to be associated with a number of health indicators. Fatigue and weakness are common consequences of anemia. Several cross-sectional studies in the elderly persons have reported the association of anemia with functional disability and poorer physical performance [2], decreased muscular strength [3], fall injury events at home [4], and increased frailty risk [5]. Two longitudinal studies suggested that elderly persons with anemia are at increased risk of physical decline and recurrent falls [6], [7]. Anemia can thus have a relevant effect on healthcare needs and, with the increasing rate of growth of the elderly population, become a significant healthcare burden [8], [9].

The hypoxic condition caused by anemia may not only negatively affect physical function but also the cognitive performance, mood, and quality of life (QoL) of the elderly person. Very few studies in community-dwelling elderly persons have explored the relationship of anemia with cognitive performance or mood, and none with QoL. Moreover, those few studies did not exclude moderate to severe anemic individuals from the analyses whose scores likely affected the results.

The main aim of the study was to investigate the association of mild grade anemia with significant health-related variables such as cognitive performance, functional status, mood, and QoL in a sample of community-dwelling elderly persons.

## Methods

### Study population

“Salute e Anemia” (“Health and Anemia”) is an observational study of all 65–84 year old individuals (N = 10,110) residing in the municipality of Biella, Piedmont, Italy, on May 12, 2003. In another study we have been conducting in another Italian population (The Monzino 80-plus Study) we found a very high prevalence of dementia, cognitive impairment, functional disability, and health problems in the oldest old. This prompted us to separate the investigation of the younger (65–84 years old) from that of the older (85+) persons (which began in May 2007), since the expected rate of exclusion would be quite different in the population above or below 84 years old. Of the 10,110 residents (6,146 women and 3,964 men) of 65–84 years old on the prevalence day, 1,131 could not be traced by phone, 80 died before being contacted, 4,398 refused to or could not donate a blood sample, and 4,501 agreed to take part. Individuals with blood tests available (mean age 73.6 years, standard deviation [SD] = 5.2) were on average approximately one year younger than than individuals without blood tests available (mean age 74.8 years, SD = 5.5) and the proportion of women was similar (60.1% and 61.2% respectively).

All elderly individuals with blood test results who gave their consent were considered for inclusion in this part of the study. Based on the information collected by the nurses to ascertain the presence of present and past diseases and level of education, individuals with neurological (stroke, epilepsy, Parkinson disease, dementia, multiple sclerosis, and other neurodegenerative diseases) or psychiatric diseases (e.g. major depression and psychotic disorders), severe sensory deficits, renal insufficiency, severe organ insufficiency (heart, lung, or liver insufficiency severe enough to limit the patient's autonomy), terminal illness (life expectancy <6 months when known), hospitalization, institutionalization, and illiteracy were excluded. Individuals with a mild grade renal insufficiency were considered eligible for the interview but were not included in the primary analyses. Purpose of these criteria was to exclude those major medical conditions already known to be associated with decreased cognitive performance, functional ability, mood, or QoL, and those individuals not reliably testable. All eligible individuals with mild anemia and a randomly selected sample of non-anemic controls were included in the study. Since secondary aim of the study was to examine the association between anemia and cancer, all eligible non anemic individuals with a past or present diagnosis of cancer were also included in this part of the study.

On average, 46 days after the blood sample collection by the nurses, a thorough home interview was conducted by trained psychologists to collect information on socio-demographic characteristics, habits, physical, social and recreational activities, and current drug use, use of health services over the past 12 months (hospital admission, emergency room, medical and instrumental investigations). The information collected by the psychologists was blinded to that previously gathered by the nurses and the two interviews were used to control for the consistency of the medical histories reported by the participants. Using the medical information collected by the nurses, two physicians rated the comorbid disease severity of each participant on a 5-point scale developed for the purposes of the study. Definitions of rating points (from 1, no impairment, to 5, extremely severe) are very similar to those of the Cumulative Illness Rating Scale [10]. Cognitive, mood, functional and QoL measures were assessed with the tests and scales described below. Trustworthiness of the interview (i.e. the cooperativeness, consistency, and confidence of the partcipants in reporting the information) was rated by the psychologist on a 5-point scale: “very good”, “good”, “sufficient”, “insufficient”, and “difficult to evaluate”.

Study procedures were in accordance with the principles outlined in the Declaration of Helsinki of 1964 and following amendments. The local research Ethics Committee of the Azienda Sanitaria Ospedaliera of Novara approved the study. Written informed consents were obtained from each participant both prior to blood sampling and at the time of the home interview.

### Definitions of anemia and mild grade anemia

Anemia was defined in accordance with World Health Organization (WHO) criteria [11] as a hemoglobin concentration less than 12.0 g/dL in women and less than 13.0 g/dL in men. Along with most grading classification systems [12], [13], mild grade anemia was defined as a hemoglobin concentration between 10.0 and 11.9 g/dL in women and between 10.0 and 12.9 g/dL in men.

### Laboratory methods

Venous blood samples were collected from participants in a sitting position by venipuncture. CBC was determined using a SISMEX SE-2100 electronic counter (Sysmex Corporation Kobe, Japan) by the central Laboratory of Biella Hospital.

### Measurements

All tests and scales used in the study are well-known and widely used measures with good validity and reproducibility. They were adminestered and scored following standardized instructions by the trained psychologists. All scores were centrally re-assessed.

#### Cognitive performance

The Mini-Mental State Examination (MMSE) is used worldwide as a brief screening instrument designed to assess global cognitive performance, principally in the elderly population [14]. The Italian version used here [15] has been standardized in a cognitively normal elderly Italian population [16]. The Word List Memory task (score range: 0–30) assesses the ability to remember newly learned information (a list of 10 words). The Word List Recall (score range: 0–10) evaluates the delayed recall and the Word List Recognition (score range: 0–10) the delayed recognition of the 10 words previously presented in the Word List Memory task. All three of these memory tests are from the CERAD battery [17] and have been previously standardized in a cognitively normal elderly Italian population [16]. The Visual Search on Matrices of Digits (score range: 0–60) tests selective and sustained attention [18]. The Stroop Colour-Word test assesses selective attention, concentration effectiveness, cognitive flexibility, ability to suppress a habitual response for an unaccustomed one [19]. The shortened version of the Stroop test has been studied in an Italian population [20], [21]. The score (range: 0–30) is expressed in terms of interference effect of time (IET) and errors (IEE). For all tests except Stroop's IET and IEE, higher scores indicate better performance.

#### Functional ability

The basic activities of the daily living (BADL) section of the Spontaneous Behaviour Interview (SBI-BADL) assesses five domains of the basic self-care ADL: dressing, eating, walking, bathing and continence. Score ranges between 0 and 30 with the lowest score indicating no degree of dependence [22]. The Instrumental Activities of Daily Living scale (IADL) investigates more complex daily tasks such as the ability to use the telephone, to prepare meals, to handle finances, etc. [23]. The scale covers eight activities for women and five for men. Raw scores were converted to a new score indicating percentage of dependence: this new score ranges between 0 and 100% with the lowest score indicating no degree of dependence. IADL and SBI-BADL are instruments with very high inter-rater and test-retest reliability [24], [25] that have been widely used in epidemiological and clinical trial studies.

#### Mood

The Italian version of the Geriatric Depression Scale (GDS) was devised to rate depression in the elderly persons [26], [27]. GDS-10 is a 10-item version highly correlated with the original and showing, with a cut-off of 3/4, a good sensitivity and specificity for significant depressive symptomatology [28]. Score ranges between 0 and 10 points, with 0 indicating absence of symptoms.

#### Quality of life

The Short-Form health survey (SF-12) contains 12 questions that were selected using statistical techniques as a subset of the SF-36 items, one of the most widely used QoL instruments in the USA and Western European countries [29]. SF-12 assesses physical functioning, role limitations due to emotional, health problems, and mental health as well as health concepts like bodily pain, general health, vitality, and social functioning. The 12-item version of the summary scales (physical and mental components) correlates with the SF-36 version in the 0.94–0.97 range. Both summary scores (SF-12 Physical and SF-12 Mental) are standardized to have a mean of 50 and standard deviation of 10 with higher scores indicating better health perception. A standardized Italian version of the questionnaire has been validated in the context of an international project [30], [31]. To further illustrate the different effect of mild anemia on the two summary scores, each individual with a score lower than 40 (that is 10 points lower than the expected value on the general population, corresponding to one unit of standard deviation) was classified as a person with a clinically relevant decrement in self-perceived QoL. For scales derived from the SF-36 Health Survey, a difference of 5 points is generally considered clinically meaningful [32], [33]. The Functional Assessment of Cancer Therapy-Anemia (FACT-An) is a 47-item questionnaire (score range: 0–188), consisting of a 27-item general questionnaire (FACT-General or FACT-G Total, score range: 0–108) measuring physical, social/family, emotional, and functional well-being and a 20-item anemia questionnaire (FACT-An Anemia subscale, score range: 0–80) that measures 13 fatigue-associated items (FACT-An Fatigue subscale, score range: 0–52) and 7 non-fatigue-associated items [34]. Each of these measures is scaled with low scores indicating poor QoL. The equivalent Italian version of the FACT was used in the present study [35].

### Statistical analysis

Simple randomization was used to select those among the non anemic elderly individuals to include in the study sample. Due to the inclusion criteria, all analyses had the diagnosis of cancer as a covariate. Differences on demographic and clinical characteristics between anemic and non anemic individuals were tested by means of linear logistic regression. Since the study population chosen to assess the association with cognitive, functional, mood, and QoL variables had no major cognitive impairment and health disorders, IADL and SBI-BADL were distributed in a highly asymmetrical way, i.e. the great majority of elderly persons had few or no problems in the activities of daily living. In absence of an agreement on a clinically relevant cut-off for this type of population, individuals were *a priori* divided between those who had no basic ADL disability and those who had, and between those who had no or almost no deficit in IADL (less than or equal to a 5% disability) and those who had a minor or major disability. Slight changes of IADL cut-off had effects on the estimated values of the association and the results are reported as secondary analysis.

Mild anemia association with each dependent variable was tested in three hierarchically related models. First, mild anemia was assessed together with oncological status, using multivariable logistic (for dichotomous variables) or linear (for numerical variables) regressions. Second, regression models were constructed to look for the effect of mild anemia on each dependent variable correcting for oncological status, age, sex, education and depressive symptoms. With the exception of SF-12 Physical, QoL values were corrected only for age, sex and education, because several of the scale questions are directed at investigating the presence of depressive symptoms. The third model was used to further correct for the presence of comorbid diseases. Thus, hypertension, heart failure, myocardial infarction, diabetes, respiratory failure, and neurologic diseases were tested in multivariable regression models, together with variables used in model 2, to ascertain if they could influence the studied associations. To control for the possible confounding effect of comorbid disease severity, the severity rating was also tested together with oncological status, age, sex, education, and depression (where relevant) in multivariable models.

To investigate suggestions in the literature on raising the normal lower limit of hemoglobin concentration [36], we next used cut-offs for hemoglobin concentration (<12.2 g/dL in women and <13.2 g/dL in men) slightly higher than those of WHO criteria (<12.0 g/dL in women and <13.0 g/dL in men) to define anemia and thus re-assigned the same elderly individuals to the mild anemic or non-anemic groups accordingly to assess the impact of mild anemia.

All *p*-values are two tailed. Data analysis was performed by using JMP v. 6.0.3 and SAS software, version 8.2 (both SAS Institute Inc., Cary, North Carolina).

## Results

**Figure 1:**
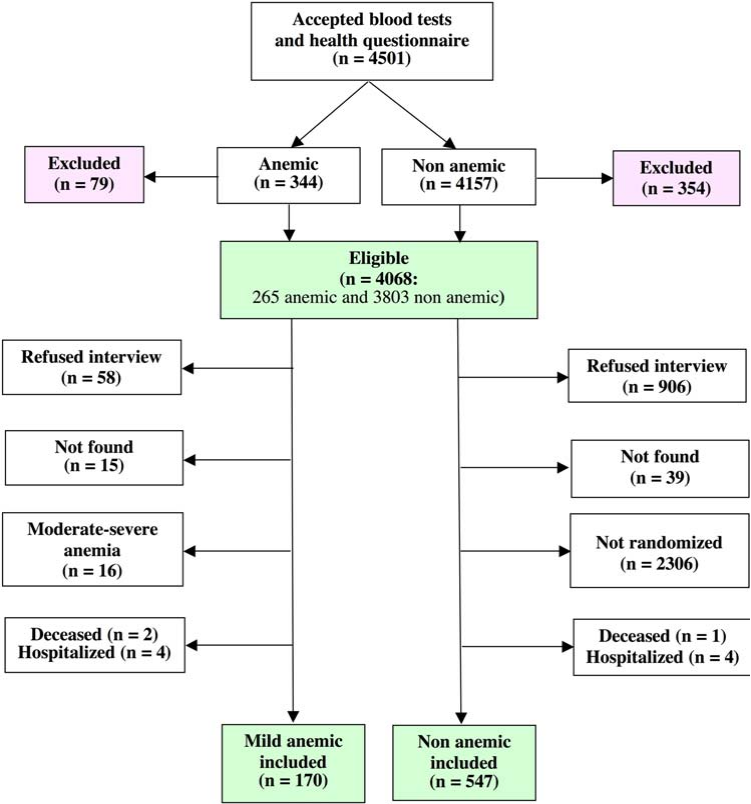
Flow chart of the study.

Figure 1 shows the flow diagram of the study. Among individuals with blood tests available, 79 (23.0%) anemic individuals (neurologic diseases: 31; renal insufficiency: 19; severe organ insufficiency: 8; severe sensory deficits: 6; institutionalized: 13; illiterate: 2) and 354 (8.5%) non anemic individuals (neurologic diseases: 201; renal insufficiency: 19; severe organ insufficiency: 27; severe sensory deficits: 62; institutionalized: 37; illiterate: 7; bedridden: 1) met exclusion criteria. A similar percentage of anemic (21.9%) and non anemic (23.8%) individuals refused the interview, while 3,104 elderly persons accepted to participate to the present study (response rate = 76.3%). Fifteen anemic and 39 non anemic elderly persons could not be found. In the anemic group a further 16 individuals were not included because affected by a moderate to severe anemia, leaving 170 mild anemic participants together with 547 randomised non anemic individuals available for the analyses.

To control for possible differences on demographic and clinical characteristics, elderly individuals included (n = 717) were compared to those not included (n = 3,351) for age, sex, education, myocardial infarction, angina, hypertension, heart failure, diabetes respiratory failure, neurologic disorders, and comorbid disease severity. Individuals not included comprised a higher proportion of women (+8.7%) and a lower proportion of individuals with a history of myocardial infarction (−1.8%) and were on average less educated (- 0.5 years) than the individuals included. As women are less affected by ischemic heart diseases and, for the generation under investigation, less educated than men, these variables are associated.

Characteristics of mild anemic and non anemic individuals included in the impact study (Table 1) reflect those of the larger groups from which participants were drawn, except for the higher prevalence of cancer in the non anemic group and, in both groups, the absence of renal failure or the drop in prevalence of those disorders like neurologic diseases because of the inclusion/exclusion criteria adopted.

**Table 1:** Characteristics of mild anemic (WHO criteria) and non anemic individuals included in the impact study

Variable	Non anemic	Mild anemic	*p* values*
Participants, n	547	170	
Mean age (SD), years	72.9 (5.1)	74.4 (5.7)	.0001
Women, %	53	54	.9110
Mean education (SD), years	8.4 (3.9)	7.6 (3.9)	.0149
Living alone, %	25.7	27.8	.6797
Smoker, current, %	11.4	13.0	.3941
Smoker, former, %	39.0	31.7	
Smoker, never, %	49.5	55.3	
Current alcohol use, %	75.0	69.8	.2733
Mean body mass index (SD), kg/m^2^	25.0 (4.0)	24.3 (3.9)	.1897
Mean hemoglobin (SD), g/dL	14.3 (12)	11.7 (7)	<.0001
Mean systolic blood pressure (SD), mmHg	143 (19)	143 (20)	.5225
Mean diastolic blood pressure (SD), mmHg	81 (10)	77 (11)	<.0001
Hypertension, %	55.2	51.8	.9459
Myocardial infarction, %	5.9	7.7	.5222
Heart failure, %	4.0	8.2	.0957
Diabetes, %	7.5	14.7	.0073
Respiratory failure, %	5.3	10.0	.0235
Neurologic diseases, %	1.7	5.9	.0189
Cancer, %	46.4	13.5	
Mean disease severity (SD)	1.20 (0.2)	1.23 (0.2)	.0191
Hospitalization (past year), %	13.7	21.2	.0213

* Adjusted for oncological status.

Mean duration of the interview was about 1.5 hours. Trustworthiness of the interviews was rated as “good” or “very good” in 88% of the cases, while in no case was the interview judged “insufficient” and in only one case (0.1%) as “difficult to evaluate”. Agreement between comparable items of the medical histories taken by the nurses and by the psychologists was very high (Cohen's κ between 0.84 and 0.93).

Table 2 shows the results of mild anemic versus non anemic group on cognitive, functional, mood and QoL variables. With the exception of basic activities of daily living (SBI-BADL) and the mental component of SF-12, in “univariate” analyses controlling for oncological status (model 1) non anemic had significantly better results than mild anemic individuals on all the other variables (*p* between .0472 and <.0001). When adjusted for age, sex, education, and GDS score (model 2), all differences between groups on cognitive (except on Visual Search on Matrices of Digits), functional, and mood variables were no longer significant. When further adjusted for the presence of comorbid conditions (model 3), only the performance on Visual Search on Matrices of Digits (least square mean difference, 95%CI = −1.7, −3.1 to −0.3), FACT-An Anemia (least square mean difference, 95%CI = −1.5, −2.9 to −0.03) and FACT-An Fatigue (least square mean difference, 95%CI = −1.4, −2.6 to −0.3) continued to be significantly worse in the mild anemic group, while the Stroop IEE (*p* = .057) and SF-12 Physical (score <40) (*p* = .067) approched statistical significance. When, instead of comorbid diseases, comorbid disease severity was entered in model 3, a further significant difference was found on SF-12 Physical (score <40): odds ratio (OR) = 1.6, 95% CI = 1.01–2.6. If the clinically-relevant cut-off on IADL score had been set up at less than or equal to 10% disability (post-hoc analysis), then mild anemia would have been significantly associated with IADL disability (fully adjusted model 3: *p* = .029).

**Table 2:** Impact of mild grade anemia (WHO criteria) on cognitive, functional, mood, and quality of life variables in elderly individuals (65–84 years)

Variable*	Non anemic	Mild anemic	Model 1†	Model 2†	Model 3†
	(n = 547)	(n = 170)	*p* values	*p* values	*p* values
Mini-Mental State Examination (0–30)	27.1 (1.8)	26.7 (2.0)	.0088	.2234	.1529
Word List Memory (0–30)	15.9 (4.4)	14.8 (4.1)	.0025	.2726	.1224
Word List Recall (0–10)	5.0 (2.0)	4.6 (2.0)	.0131	.5014	.3408
Word List Recognition (0–10)	8.6 (1.7)	8.2 (1.9)	.0105	.2523	.1573
Visual Search on Matrices of Digits (0–60)	47.3 (8.4)	44.3 (9.6)	<.0001	.0217	.0213
Short Stroop Colour-Word IEE (30-0)	0.9 (2.1)	1.5 (3.0)	.0053	.0672	.0566
Short Stroop Colour-Word IET ( -0 sec.)	36.0 (19.1)	40.7 (24.8)	.0130	.2404	.2766
Short Stroop Colour-Word worst quartile	21.9%	32.1%	.0117	.1204	.1544
Geriatric Depression Scale (10-0)	1.8 (2.2)	2.2 (2.4)	.0146	.1150	.3303
Geriatric Depression Scale score ≥4	17.9%	26.0%	.0162	.1009	.2256
IADL (% with a disability >5%)	11.2%	20.1%	.0052	.1837	.1966
SBI-BADL (% with at least 1 minor disability)	21.8%	20.0%	.9920	.3631	.2747
SF-12 Physical (0–100)	47.3 (8.7)	45.3 (10.0)	.0014	.0484	.1650
SF-12 Physical score <40	19.5%	29.9%	.0014	.0219	.0665
SF-12 Mental (0–100)	51.8 (9.1)	52.5 (8.6)	.3996	.1680	.0991
SF-12 Mental score <40	11.3%	9.2%	.4254	.2066	.1323
FACT-An (0–188)	141.0 (18.3)	136.7 (21.5)	.0053	.0542	.1770
FACT-General (0–108)	75.8 (12.2)	73.8 (12.9)	.0472	.2346	.4003
FACT-An Anemia (0–80)	65.1 (7.8)	62.7 (10.2)	.0003	.0058	.0456
FACT-An Fatigue (0–52)	43.4 (5.8)	41.5 (7.7)	<.0001	.0014	.0109

* Between brackets score ranges with best scores on the right.

† Model 1 includes mild anemic and oncological status only; Model 2 includes Model 1 plus age, sex, education, and (except: Geriatric Depression Scale, SF-12 Mental, and FACT scores) Geriatric Depression Scale score; Model 3 includes Model 2 plus hypertension, diabetes, heart failure, myocardial infarction, respiratory failure, neurologic disorders.

If instead of all, only the expected 11.2% of the elderly with a past or present diagnosis of cancer were randomly included into the control group (that is recreating a simple random sample from the non anemic population), the results would not change: in the fully adjusted model mild anemia would remain significantly associated with the same cognitive and QoL variables above reported and also with Stroop IEE measure (*p* = .043). If the 19 individuals with a mild grade renal insufficiency (11 mild anemic and 8 non anemic) were also included into the analyses, beyond the above significant variables, in model 3 adjusted for comorbid diseases also Stroop IEE (*p* = .049) and in model 3 adjusted for comorbid disease severity also Stroop IET (worst quartile) (*p* = .034) and IADL (*p* = .020) would reach statistical significance, while several other variables would approach statistical signifincance.

If the lower limit of normal of hemoglobin concentration is raised to define anemia in women (<12.2 g/dL) and men (<13.2 g/dL), non anemic individuals (*n* = 531) tend to perform slightly better and mild anemic (*n* = 186) slightly worse than the corresponding groups defined using WHO criteria and the differences between groups tend to increase reaching statistical significance in the adjusted model 3 also for Word List Recognition, Short Stroop IET (worst quartile), and SF-12 Physical (mean score and score <40) (Table 3).

**Table 3:** Impact of mild grade anemia (hemoglobin concentration: women, 10.0–12.1 g/dL; men, 10.0–13.1 g/dL) on cognitive, functional, mood, and quality of life variables in elderly individuals (65–84 years)

Variable*	Non anemic	Mild anemic	Model 1†	Model 2†	Model 3†
	(n = 531)	(n = 186)	*p* values	*p* values	*p* values
Mini-Mental State Examination (0–30)	27.1 (1.8)	26.7 (2.0)	.0055	.1681	.1069
Word List Memory (0–30)	15.9 (4.4)	14.8 (4.1)	.0018	−2136	.0733
Word List Recall (0–10)	5.1 (2.0)	4.6 (2.0)	.0034	.2389	.1391
Word List Recognition (0–10)	8.6 (1.6)	8.2 (1.9)	.0014	.0696	.0383
Visual Search on Matrices of Digits (0–60)	47.4 (8.4)	44.2 (9.4)	<.0001	.0061	.0060
Short Stroop Colour-Word IEE (30-0)	0.9 (2.1)	1.5 (2.9)	.0087	.0997	.0752
Short Stroop Colour-Word IET ( -0 sec.)	35.6 (19.0)	41.4 (24.4)	.0013	.0573	.0606
Short Stroop Colour-Word worst quartile	20.9%	34.2%	.0005	.0113	.0150
Geriatric Depression Scale (10-0)	1.8 (2.2)	2.3 (2.4)	.0036	.0440	.1637
Geriatric Depression Scale score ≥4	17.5%	26.5%	.0068	.0572	.1551
IADL (% with a disability >5%)	10.9%	20.0%	.0033	.1864	.2042
SBI-BADL (% with at least 1 minor disability)	21.5%	21.3%	.6089	.6284	.5329
SF-12 Physical (0–100)	47.6 (8.5)	44.9 (10.1)	<.0001	.0050	.0295
SF-12 Physical score <40	18.6%	31.7%	<.0001	.0029	.0128
SF-12 Mental (0–100)	51.9 (9.0)	52.2 (9.7)	.6422	.2885	.1847
SF-12 Mental score <40	11.1%	10.0%	.6874	.3716	.2610
FACT-An (0–188)	141.2 (18.1)	136.3 (21.6)	.0014	.0204	.0830
FACT-General (0–108)	75.9 (12.1)	73.7 (13.0)	.0263	.1607	.2942
FACT-An Anemia (0–80)	65.3 (7.6)	62.5 (10.3)	<.0001	.0008	.0099
FACT-An Fatigue (0–52)	43.5 (5.6)	41.4 (7.8)	<.0001	.0003	.0032

* Between brackets score ranges with best scores on the right.

† Model 1 includes mild anemic and oncological status only; Model 2 includes Model 1 plus age, sex, education, and (except: Geriatric Depression Scale, SF-12 Mental, and FACT scores) Geriatric Depression Scale score; Model 3 includes Model 2 plus hypertension, diabetes, heart failure, myocardial infarction, respiratory failure, neurologic disorders.

## Discussion

The findings of the present cross-sectional study suggest an independent associaton of mild grade anemia with worse selective attention performance and disease-specific QoL ratings in the elderly persons living in the community.

The association of anemia with cognitive status has been investigated in very few community-based studies. Elwood et al. did not find evidence of a significant correlation between hemoglobin concentration and four tests of memory in a subsample of 164 selected and unspecified elderly individuals, very few of whom (10%?) must have been anemic [37]. Recently, Denny et al. found significantly worse baseline scores and greater decline four years later on a 2-minute global screening test for dementia (the Short Portable Mental Status Questionnaire) among anemic household elderly persons, but these findings were rather inconsistent in women and men and in the African American and Caucasian subsamples [38]. Literature on the association between anemia and dementia is limited and the results inconsistent [39]–[44]. In the present study, mild anemic performed worse than non anemic elderly individuals on all tests of cognitive function. However, as in the Honolulu-Asia Aging Study [45], most differences were no longer significant once adjusted for demographic and clinical confounding factors. Only selective attention continued to be significantly associated with mild anemia in the fully adjusted model 3 and this finding goes along with the results reported by Chaves et al. who found in community-dwelling highly educated elderly women that the likelihood of being in the worst tertile of the Trail-Making Test performance was higher for the 30 women with mild anemia [46].

Functional disability was found to be significantly associated with anemia of any grade in three community-dwelling studies [2], [38], [47], though anemia was only self-reported in one [47]. Instead, no significant differences in instrumental and basic ADL were found between anemic and non anemic elderly persons in a Japanese population living in the community [48]. As expected for non demented elderly individuals with no major health problems like those included in the impact study, instrumental and basic ADL were quite well preserved and thus no significant differences could be evidenced in the fully adjusted model 3 between mild anemic and non anemic groups. Post-hoc analysis seems to suggest that higher level of IADL disability could be associated with mild anemia.

To our knowledge, only two community studies have examined the relationship between anemia and depression: in an elderly Italian population, Onder et al. reported a significant link between anemia of any severity and depressive symptoms [49], while Ishine et al. did not find a significant difference in an elderly Japanese population [48]. In the present study we could not demonstrate an association of mild anemia with the presence of depressive symptomatology independent of the influence of other potential confounders.

Apart from selected populations of patients with cancer-related, end-stage renal disease-related and chronic obstructive pulmonary disease-related anemia [50]–[52], no study investigated the possible effect of anemia or mild anemia on QoL in elderly community-dwelling persons. Using well-validated instruments, in the present study mild anemia was found to be significantly associated with disease-specific measures of QoL. These findings are consistent with recent results of a crossover clinical trial in 62 elderly patients with chronic anemia where FACT-An anemia and fatigue subscales showed sensitive measures of QoL change over a 32-week study period [53].

Following suggestions from the literature [6], [36], [54], [55], we also examined the effect of mild anemia on several variables when anemia was defined as a hemoglobin concentration just higher (+0.2 g/dL) [36] than that of WHO criteria. It is beyond the scope of the present study to discuss the predictive validity of the various cut-offs of hemoglobin concentration suggested to define anemia in the elderly population, but it is interesting to note that, since the elderly persons with a hemoglobin concentration just above the traditional WHO lower limit of normal, however feebly, worsened the mean performance of the non anemic group on almost every variable, the somewhat arbitrary WHO cut-off chosen did not bias the estimated effect of mild anemia on the various variables reported in Table 2, but rather yielded conservative results.

Some potential limitations should be acknowledged. Information on comorbid diseases mainly relied on the individual self-report, but the clinical pictures reported by the elderly persons have been shown to be accurate and complete [56]–[58], and, in the present study, the reliability of the interview was very high. Moreover, since imprecise reporting would likely apply to both mild anemic and non anemic groups, any inaccuracy would result in an underestimation of the associations. Although the possible influence of a non-response bias on the associations studied cannot be excluded, this seems rather improbable considering the condition examined (an anemia of mild grade of which most of the elderly persons were unaware) together with the nature of the dependent variables studied. Residual confounding by other clinical conditions may not have been recognized, even though the large number of confounders entered in multivariable analyses may have more likely led to overadjustment and consequent underestimation of the strength of the associations. The cross-sectional and observational nature of the study does not permit inferring either a causal relation between mild anemia and dependent variables or a reduction of the risk once mild anemia is treated.

Mild anemia is frequent and mostly overlooked in the elderly population. Our findings show that mild grade anemia is independently associated with worse selective attention performance and disease-specific QoL ratings. Longitudinal studies would further increase our knowledge of the potential risks of mild anemia to the health of the elderly persons, while controlled clinical trials could investigate whether treating this condition would reduce the associated risks.
